# Gender-related differences in the multi-pathway effect of social determinants on quality of life in older age—the COURAGE in Europe project

**DOI:** 10.1007/s11136-017-1530-8

**Published:** 2017-03-03

**Authors:** Beata Tobiasz-Adamczyk, Aleksander Galas, Katarzyna Zawisza, Somnath Chatterji, Josep Maria Haro, José Luis Ayuso-Mateos, Seppo Koskinen, Matilde Leonardi

**Affiliations:** 1 Department of Medical Sociology, Chair of Epidemiology and Preventive Medicine, Jagiellonian University Medical College, Kopernika 7a, 31-034 Krakow, Poland; 2 Department of Epidemiology, Chair of Epidemiology and Preventive Medicine, Jagiellonian University Medical College, 7, Kopernika St, 31-034 Krakow, Poland; 3 Department of Health Statistics and Information Systems, World Health Organization, 20 Avenue Appia, 1211 Geneva 27, Switzerland; 4 Instituto de Salud Carlos III, Centro de Investigación en Red de Salud Mental, CIBERSAM, Madrid, Spain; 5 Parc Sanitari Sant Joan de Déu, CIBERSAM, Universitat de Barcelona, Sant Boi de Llobregat, Dr. Antoni Pujadas, 42, 08830 Sant Boi de Llobregat, Barcelona, Spain; 6 Department of Psychiatry, Hospital Universitario de La Princesa, Instituto de Investigación Sanitaria Princesa (IP), Madrid, Spain; 7 Department of Psychiatry, Universidad Autónoma de Madrid, Arzobispo Morcillo, 4, 28029 Madrid, Spain; 8 National Institute for Health and Welfare, Mannerheimintie, 166, 00300 Helsinki, Finland; 9 Fondazione IRCCS, Neurological Institute Carlo Besta, Via Celoria, 11, 20133 Milano, Italy

**Keywords:** Gender-related differences, Social networks, Social participation, Social support, Loneliness, Trust, Quality of life

## Abstract

**Purpose:**

Gender-related differences in life expectancy, prevalence of chronic conditions and level of disability in the process of ageing have been broadly described. Less is known about social determinants, which may have different impacts on quality of life in men and women. The investigation aims to reveal gender-related differences in social determinants on quality of life assessed by a multi-pathway model including health, social, demographic and living place characteristics.

**Methods:**

The study group consisted of 5099 participants aged 50+ representing general populations of three different European regions (Finland, Poland, Spain) who participated in COURAGE in EUROPE Project. Standardized tools were used to measure quality of life (WHOQOL-AGE) and social determinants (COURAGE Social Network Index, OSLO-3 Social Support Scale, UCLA Loneliness Scale, participation scale and trust). A multipath model considering exogenous predictors (demographic, economic), mediators (social) and endogenous outcome (QOL) was created to reveal the role of determinants. Gender-related differences were investigated across three age categories: 50–64; 65–79 and 80+.

**Results:**

The model (RMSEA = 0.058; CFI = 0.939) showed the effects of all of the investigated determinants. Gender-related differences in the association between social constructs and QOL were observed for social networks in the group of 80+, for social support in the group of 50–64 and 65–79 years, and for social participation in the group of 65–79 years. Males benefited more (in QOL) from social networks and social support, and women from social participation.

**Conclusions:**

The research provides valuable knowledge about the role of social determinants in QOL considering complex relations between different social constructs. Additionally, the results showed gender-related differences in the associations between social networks, social support, social participation and QOL, suggesting that men might benefit more from the interventions in the first two. Although our research did not investigate the effects of interventions, the results show directions for future investigations, how to shape social interventions at the population level to improve quality of life of older adults, and thus help achieve successful ageing.

**Electronic supplementary material:**

The online version of this article (doi:10.1007/s11136-017-1530-8) contains supplementary material, which is available to authorized users.

## Introduction

Gender-related differences in the process of ageing have been well documented in relation to life expectancy, prevalence of chronic conditions, level of disability and functional status, supporting a well-known paradox that men are likely to die earlier than women, but older women suffer from higher level of chronic health conditions and disability [1]. Still, less is known about psychosocial dimensions of older life which could influence the successful ageing as a consequence of life experiences coming from the previous stages of the life course.

On the one hand, studies of the relation between gender and ageing have focused on changes in the marital status, social roles and relationships, especially due to feminization of older part of society. The domination of older women in the social structure in later age groups, mostly widowed or divorced, living alone could be associated with higher risk of poorer quality of life [1–3]. On the other hand, the last decades have seen a significant increase of research interest in differences between men and women in the ageing process [4].

In public health, successful ageing is generally understood as the ability to maintain high level of quality of life in older ages. Von Faber defined it as the optimal state for well-being including good quality of life and satisfaction with the present life [5]. Litwin further developed this definition to include the ability to remain integrated within social life [6], a concept which has led to an increase in research interest in the determinants of successful ageing. Existing data showed some opposite results: while Arisa-Merino [7] found a higher proportion of successfully ageing men (18.4%) in comparison to women (9.2%), the longitudinal British cohort study [8] demonstrated 12.8% men and 14.6% women successfully ageing. Documented data also confirmed different predictors of successful ageing in men and women [8, 9].

Psychosocial theories and concepts used for the explanation of gender-related differences in the older stages of life are usually based on life course approach (convoy model of social relations, psychosocial and material resources such as psychological, social, financial well-being and security gathering during the life-span, social inequalities). Such determinants were found to significantly influence adaptation to changes attendant upon the process of ageing related to social networks’ structure and specific social ties as well as the various coping strategies with stressful life events experienced by men and women [3, 10–12].

Different definitions of quality of life have been developed over the last decades, and significant evolution from objective to subjective indicators as well as in measuring specific dimensions has been observed in that multifaceted concept. Definitions have focused on subjective, individual’s perception of the quality of life, as developed by WHO: *Quality of Life as individuals’ perception of their position in life in the context of the culture and value systems in which they live and in relation to their goals, expectations, standards and concerns* [13], which has also been used as a basic theoretical perspective for an assessment of quality of life in older age [14].

## Theoretical framework

Taking into account the broadness of the definition of quality of life and different concepts of successful ageing, several contributing factors have been identified [15–18]. Recently, Jopp stressed the role of social resources such as informal social network, social support, social participation (formal social network), feeling of social belonging as well as activities including work activity, sport, travel, hobbies, volunteering, attitudes and beliefs about life as the components of successful ageing [19].

### Social networks

The *c*oncept of social network has been developed over the last decades, based on social integration theory since Durkheim’s classic work [20–22]. The conceptual model links social networks to health outcomes (health-related quality of life), from social structure conditions at macro level (such as culture, socioeconomic factors, politics and social change), and shows the role of social networks mainly in providing opportunities for psychosocial mechanisms at micro level, such as social support, social engagement and access to material goods, which in turn influence health through health behavioural pathways, psychological pathways (self-efficacy, self-esteem, depression/distress, sense of well-being) and physiological pathways (immune system functions, cardiovascular reactivity, cardiopulmonary fitness, transmission of infectious disease) [20]. Most common perspectives define the term “social network” as the web of identified social relationships that surrounds an individual person, characteristics of those linkages and the individual’s perception of them [23, 24] or channels through which pragmatic help as well as emotional and psychological support can be exchanged between individuals [25]. Characteristics of network are usually based on ties (strength, frequency of contact, duration, reciprocity and intimacy) and network’s features (size, density, degree, boundedness, proximity, homogeneity) [20, 21].

Several theoretical frameworks as well as methodological approaches have been proposed to explain the role of social relationships—especially characterized by closeness—in general well-being, mental health and other aspects of health-related quality of life. Many well-documented studies investigated the relationship between social relations (considering either main or buffering effect) and mortality [6, 26–32]. Special attention has been paid to the relationship between health status of elders and their participation in different social networks as a prevention strategy against social disintegration and exclusion, as well as against loneliness [33–41] and the relationship between social support (as a role of social networks) and subjective well-being across age [42]. Gallegos-Carillo observed that older persons with depressive symptoms had the lowest scores in all dimensions of health-related quality of life (HRQoL) [43]. In the US, Fiori explored cultural differences in the profiles of social relations (structural, functional and qualitative aspects) as well as the role of social network types in mental and physical health [44]. In addition, she observed two types of “friend-focused” networks (supported and unsupported) and two types of “restricted networks” (structurally and functionally restricted) and in Japan “married and distal”. Fiori paid special attention to restricted (socially isolated) network type which was found to be related to lowest well-being in the US. However, this factor was not confirmed for Japan, suggesting the existence of cross-cultural determinants [44]. Differences among one sex (older women) in prospective study of the association between living arrangement and emotional well-being did not confirm that women living independently suffered from higher risk of social isolation and decline in functional status, because contacts with friends and relatives and the level of social engagement were significant protective factors [18].

### Social support

#### Social support coming from social network

The hierarchical compensatory model showed that older people have a rank-ordered preference for receiving social support from others (firstly turn to family members: spouse, children, grandchildren, other relatives, friends and professionals) expecting instrumental, emotional and financial support [4]. Intergenerational solidarity model developed by Bengtson (2002) [45] explained not only directions of social support associated with caregiving to older parents but also psychosocial benefits.

The model developed by Kawachi and Berkman [46] shows several pathways which can affect psychological well-being through participation in social networks.

Most of the studies confirmed the health-promoting effect of social support; moreover, an absence of negative social interactions may be more important for mental health than the presence of supportive interactions. Schuster showed that negative interactions with spouse, relatives and friends are more predictive of depressed mood than supportive interactions (especially with spouse and friends) [47]. Finch et al. found that the positive and negative social ties among older adults were independent domains of social experiences [48]. Positive social ties were related to psychological well-being and the negative ones were predictive of both psychological well-being and distress [48]. These results demonstrated the importance of assessing both positive and negative aspects in explaining the psychological adjustment of older adults.

### Trust

Interpersonal trust represents how individuals manage their collective actions for mutual motives and objects. People need trust in order to be able to interact with others. In social networks, trust must be reciprocal and must include important interpersonal psychological qualities that strengthen its significance. Solidarity between people is a natural prerequisite for social trust [49].

### Loneliness

Most of the studies stress the role of loneliness as a consequence of changes in social roles and limitation in social relationships as a significant determinant of the quality of life at the older stages of life.

Loneliness has been defined as a state opposite to the strong social ties and social networks or as a consequence of weak social ties. From the dynamic perspective, loneliness is precipitated by changes in a person’s social relationships that lead to a sub-optional level of achieved social interactions. These changes may effect a single relationship or may affect a person’s total network of social relations. Social loneliness and social isolation in older age have been described using objective and subjective measures. Victor et al. showed the role of loneliness, social isolation and living alone in relation to successful ageing and quality of life in older life, and mentioned that the concept of loneliness has been interpreted in different ways using several theoretical explanations of the cause of loneliness. The interactionist theory, based upon the attachment theory of Bowlby, combines the individual emotional aspects with social aspects [24]. Following this perspective, loneliness was caused by a combination of the lack of an attachment figure and the absence of an adequate social network and the experience of loneliness was dependent on the individual’s personality type [24, 50].

### Social participation/social engagement

Social and civic participation focuses on voluntary associations with or without identification, participation in a group or community, free chosen groups, organizations, clubs, neighbourhood and communities. Voluntary social participation increases self-esteem, reduces depression and distress, and improves the sense of personal happiness and well-being [49].

Classical studies focusing on differences between men and women in relation to social characteristics confirmed differences in life style, quality of social networks, social support and strategies of coping with stressful life events. Mortality studies also confirmed different levels of risk of death between men and women in relation to social networks and health-related quality of life, but the explanation of the role of social determinants in quality of life and longevity still remains unclear.

### Aim of the study

Therefore, the aim of this investigation was to reveal gender-related differences in social determinants on quality of life assessed by a multi-pathway model considering health, social, demographic and living place characteristics.

## Methods

**Fig. 1:**
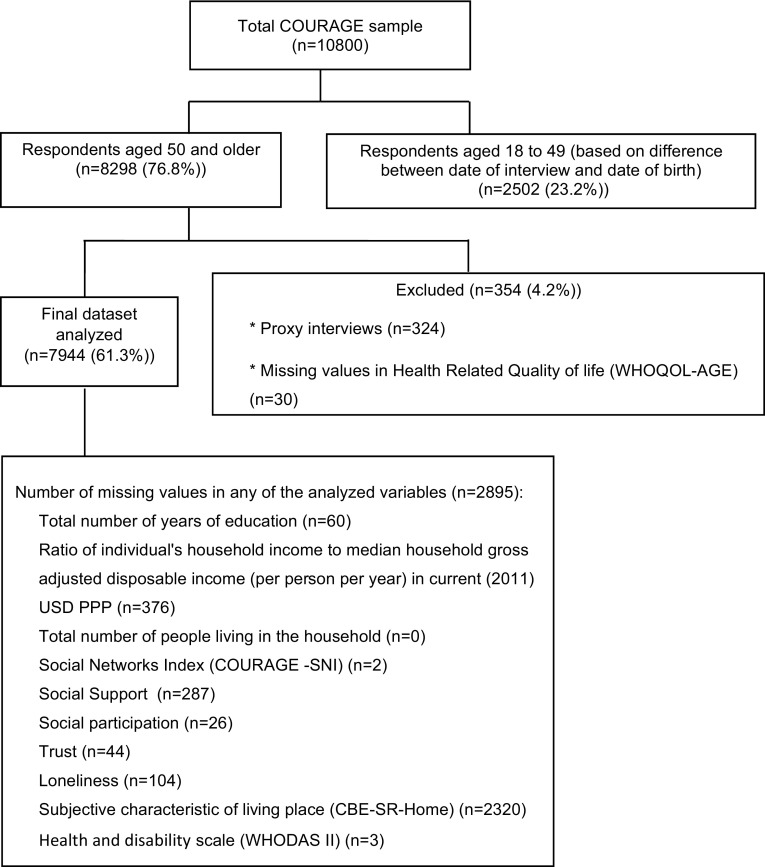
Selection of participants

The cross-sectional study COURAGE *in Europe* was conducted during 2009–2012. The field part of the study took place in 2011. Face-to-face interviews were performed at homes of individuals randomly sampled from the non-institutionalized adult population (18+) of Finland (n = 1976), Poland (n = 4071) and Spain (n = 4753) based on the multistage clustered design. The response rate was 53.4% for Finland, 66.5% for Poland and 69.9% for Spain. Countries were selected to give a broad representation across different European regions (north, Scandinavia, south, Mediterranean and central European, post-transition countries), with different populations and different health characteristics [51, 52]. The present study was approved by the Ethical Committee of Neurological Institute Carlo Besta, Milan, Italy, project coordinator; the Ethics Review Committee, National Public Health Institute, Helsinki, Finland; the Bioethical Committee, Jagiellonian University, Krakow, Poland; Ethics Review Committee, Parc Sanitari Sant Joan de Déu, Barcelona, Spain; and Ethics Review Committee, La Princesa University Hospital, Madrid, Spain. The sample available for the analysis is described in Fig. 1.

### Measurements

The construct of social networks was measured by the COURAGE Social Network Index (COURAGE-SNI) developed as a multidimensional instrument which assesses elements of function of social networks (frequency of direct contact, ties and social support) in eight structural components (spouse or partner, parents, children, grandchildren, other relatives, neighbours, friends and co-workers) (for validity properties, see [52]).

Perceived social support was measured by the OSLO-3 Social Support Scale (for validity properties, see [53]).

Social participation was assessed as a factor score of eight items measured on a five-point Likert scale ranging from *never* to *daily*. Questions concerned the frequency of attendance in public meeting, meeting with community leader, attendance at any group or organizational meeting, sport clubs, competitions or doing sport with someone else, work with people from neighbourhood to fix or improve something, having friends over at the home, visiting or hosting someone who lives in a different neighbourhood and getting out to take part in social meetings (Cronbach’s Alpha = 0.74).

Trust was measured as a factor score of five items: the first four concerned the extent of trust towards people from neighbourhood, work, strangers and members of their families, with five-point Likert scale responses (from *very great extent* to *very small extent*); in addition, one dichotomous item was related to general trust towards people (Cronbach’s Alpha = 0.72).

Loneliness was assessed by the three-item UCLA Loneliness Scale (for validity properties, see [54]).

Health-related quality of life (HRQoL) was assessed by the World Health Organization Quality of Life Assessment-Age (WHOQOL-AGE). The tool comprises 13 items focusing on areas which are important for older adults (for validity properties, see [55]).

All aforementioned scales ranged from 0 to 100 points and the results were interpreted as higher level of social network saturation, higher level of social support, social participation, trust, loneliness and quality of life.

Additionally, the following covariates were taken into consideration: health and disability—measured by the World Health Organization Disability Assessment Schedule version II (WHODAS II) and scored on a 0–100 scale with higher scores indicating greater disability; the CBE-SR-Home scale (The COURAGE Built Environment Self-Reported Questionnaire—Living Place/Home) to measure subjective characteristic of living place/home with the scale ranging from 0 to 100, and higher scores indicating that the living place is perceived as less risky and more usable [56]; ratio of individual’s household income to median household gross adjusted disposable income (per person per year) in current (2011) USD PPP; age; total number of years of education; and total number of people living in the household.

### Statistical analysis

Baseline characteristics of participants have been presented by gender across three age categories (50–64 years: *pre-elderly*; 65–79 years: *old* and 80+ understood as *old–old*). Differences across groups were tested by the Chi square test and by the *U* Mann-Whitney test as the distributions of these variables tested by the Kolmogorov–Smirnov test with Lilliefors correction were skewed.

The following groups of variables were considered to play a role in the multi-pathway effect on HRQoL: demographic characteristics (age, total number of years of education, ratio of individual’s household income to median household gross adjusted disposable income (per person per year) in current (2011) USD PPP, total number of people living in the household), besides subjective characteristic of living place/home, health and disability status and social determinants (social networks, social support, social participation, trust, loneliness). To identify important pathways between the aforementioned factors, we assessed the level of the bivariate correlation coefficients using Spearman’s rank rho.

**Fig. 2:**
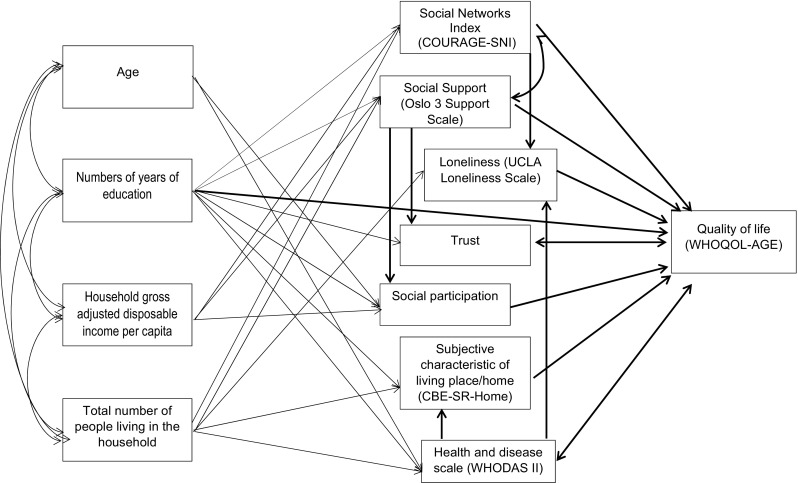
Path model specifying the association between social and demographic, living place, health-related determinants and quality of life. *Note* RMSEA = 0.058; CFI = 0.939

Two strategies were used to build the pathway model. In the first strategy, we have decided to use these pathways in which the correlation coefficients amount to at least 0.3 and then other pathways based on modification indices were set up to obtain acceptable model fit (Online resources 1, Figs. 1, 2, 3). As a second strategy, we tried to be more close to the theories than to the observations made and thus demographic and economic variables were considered as exogenous predictors, social determinants as mediators and quality of life as an endogenous outcome. The second model was conceptually easier to understand and interpret. For the second model, we started to include pathways between variables with the correlation coefficients amounting to at least 0.3, but this strategy failed to achieve acceptable model fit; therefore, we changed the correlation coefficient criterion on 0.1, which finally provided us opportunity to create a model which is the final one as presented in this manuscript (Fig. 2.). Other created models are presented in the supplementary material to this article. Models were designed based on multiple imputation of missing data using Bayesian analysis.

The goodness of fit of the model was tested using the Root Mean Square Error of Approximation (RMSEA) and Comparative Fit Index (CFI). The values of RMSEA lower than 1 and CFI higher than 0.9 indicate acceptable fit [57].

The final model was analysed to find differences in the effect of social determinants on HRQoL between age categories, gender and gender across age groups by multi-group procedure.

The descriptive analyses were carried out in IBM SPSS Statistics version 22 for Windows. The path model was implemented in MPlus (Version 7.1) using maximum likelihood estimator.

## Results

Our study included 5099 individuals aged 50+, of which 2537 (50.1%) were 50–64 years old, 1834 (35.6%) at 65–79 and 728 (14.2%) were at the age of 80+ (old–old). The average age of the population studied was 66.5 years (SD = 10.7).

The number of years of education differed between men and women in 65–79 and 80+ age categories, being higher among men. There were no differences observed in the individual’s income level expressed as a ratio of individual’s household income to median household income in a country recalculated as gross adjusted disposable income per person per year in current (2011) USD PPP.

Males on average assessed HRQoL better and had higher level of social participation than females regardless of the age group. Men had also higher level of social networks among people aged 65–79. Higher perception of loneliness was observed among pre-elderly and older women. Pre-elderly men indicated higher level of social trust and assessed their living places as more usable than women. Similarly, higher disability level was observed among women in all groups (Table 1).

**Table 1:** Baseline characteristics of participants by gender across countries

[n (%)]	50–64		P value	65–79		P value	80+		P value
	Men	Women		Men	Women		Men	Women	
	1078 (42.5)	1459 (57.5)		780 (42.5)	1054 (57.5)		253 (34.8)	475 (65.2)	
	Median (Q1; Q3)	Median (Q1; Q3)		Median (Q1; Q3)	Median (Q1; Q3)		Median (Q1; Q3)	Median (Q1; Q3)	
Total number of years of education	11.0 (9.0; 14.0)	12.0 (9.0; 14.0)	0.053	10.0 (7.0; 13.0)	8.0 (7.0; 12.0)	<0.001	7.0 (5.0; 11.0)	7.0 (5.0; 9.0)	0.001
Ratio of individual’s household income to median household gross adjusted disposable income (per person per year) in current (2011) USD PPP	0.5 (0.3; 1.0)	0.5 (0.3; 1.0)	0.848	0.5 (0.3; 0.8)	0.5 (0.3; 0.8)	0.658	0.6 (0.3; 0.9)	0.5 (0.3; 0.8)	0.084
Total number of people living in the household^1^	2.0 (2.0; 3.0)	2.0 (2.0; 3.0)	0.001	2.0 (2.0; 2.0)	2.0 (1.0; 2.0)	<0.001	2.0 (1.0; 2.0)	1.0 (1.0; 2.0)	<0.001
Health-related quality of life (WHOQOL-AGE)	72.3 (62.3; 80.9)	71.4 (61.0; 78.6)	0.001	72.3 (63.4; 79.5)	69.8 (58.4; 77.9)	<0.001	67.4 (55.6; 76.2)	62.5 (50.0; 72.8)	<0.001
Social Networks Index (COURAGE -SNI)	69.9 (60.3; 78.9)	68.8 (59.6; 77.4)	0.007	70.6 (62.5; 78.9)	67.9 (58.6; 76.9)	<0.001	67.3 (55.1; 76.1)	62.5 (53.2; 73.2)	0.009
Social support (Oslo 3 Support Scale)	72.7 (63.6; 81.8)	72.7 (63.6; 81.8)	0.881	72.7 (63.6; 81.8)	72.7 (63.6; 90.9)	0.018	72.7 (54.5; 81.8)	63.6 (54.5; 81.8)	0.229
Social participation	25.3 (12.5; 37.0)	22.6 (12.5; 34.3)	<0.001	21.4 (11.3; 34.7)	17.8 (9.4; 30.7)	<0.001	15.1 (4.8; 28.4)	10.5 (4.8; 20.6)	<0.001
Trust	55.1 (46.3; 67.0)	52.8 (42.5; 67.0)	<0.001	55.1 (43.8; 65.0)	53.4 (42.4; 65.0)	0.429	52.6 (41.7; 65.7)	50.7 (39.8; 63.0)	0.039
Loneliness (UCLA Loneliness Scale)	0.0 (0.0; 16.7)	0.0 (0.0; 16.7)	<0.001	0.0 (0.0; 16.7)	0.0 (0.0; 16.7)	<0.001	0.0 (0.0; 33.3)	0.0 (0.0; 33.3)	0.009
Subjective characteristic of living place/home (CBE-SR-Home)	82.5 (58.2; 100.0)	80.1 (53.8; 100.0)	0.066	83.3 (57.7; 100.0)	80.1 (59.1; 100.0)	0.513	73.7 (50.1; 95.0)	69.9 (49.0; 93.1)	0.185
Health and disability scale (WHODAS II)	2.8 (0.0; 11.1)	5.6 (0.0; 16.7)	<0.001	5.6 (0.0; 19.4)	11.1 (2.8; 30.6)	<0.001	19.4 (5.6; 44.4)	33.3 (13.9; 55.6)	<0.001

P value for Chi-square test or Mann–Whitney (*U*) test; SD—standard deviation; Q1—first quartile; Q3—third quartile

The strongest correlations were observed between HRQoL and health and disability scale (−0.58). The strength of correlations between HRQoL and social determinants was moderate and ranged from 0.30 for social networks to −0.36 for loneliness. Relatively strong correlations were observed for HRQoL and subjective characteristic of living place (0.38). Social network correlated positively with social support (0.51) and negatively with loneliness (−0.31). Similar value of the correlation was found between social support and trust (0.34). In addition, the strongest correlations with age were observed for health and disability scale (0.36), number of years of education (−0.41) and total number of people living in the household (−0.31) (see Table 2).

**Table 2:** Pairwise correlation coefficients between analysed variables

	1	2	3	5	6	7	8	9	10	11	12
1. Age	1										
2. Numbers of years of education	−0.41**	1									
3. Household gross adjusted disposable income per capita	0.04**	0.26**	1								
5. Total number of people who live in the household	−0.31**	−0.07**	−0.30**	1							
6. Health-related quality of life (WHOQOL-AGE)	−0.13**	0.23**	0.04**	0.08**	1						
7. Social networks index (COURAGE-SNI)	−0.08**	−0.01	−0.19**	0.29**	0.30**	1					
8. Social support (Oslo 3 Support Scale)	−0.01^*^	−0.002	0.13**	0.11**	0.32**	0.51**	1				
9. Social participation	−0.20**	0.26**	0.16**	0.02*	0.34**	0.09**	0.15**	1			
10. Trust	−0.05^*^	0.12**	0.01	0.03**	0.32**	0.26**	0.34**	0.23**	1		
11. Loneliness (UCLA Loneliness Scale)	0.07**	−0.07**	−0.04**	−0.20**	−0.36**	−0.31**	−0.21**	−0.14**	−0.19**	1	
12. Subjective characteristic of living place/home (CBE-SR-Home)	−0.06**	0.10**	0.07**	0.10**	0.36**	0.26**	0.25**	0.10**	0.13**	−0.19**	1
13. Health and disability scale (WHODAS II)	0.36**	−0.31**	−0.03^*^	−0.14**	−0.58**	−0.18**	−0.14**	−0.28**	−0.23**	0.29**	−0.24**

Note: The values of correlation coefficients used as the basis for the model presented in Fig. 2 are in bold (Online resources 1, Fig. 2)

**p < 0.01, *p < 0.05

In the final model, social networks and level of support were positively regressed on income (beta = 0.002, beta = 0.01, respectively) and on the number of people (family members in 98% of cases) living in an individual’s household (beta = 0.25, beta = 0.10, respectively); loneliness was negatively regressed on the number of people living in the household (beta = −0.07); trust was positively regressed on the number of years of education (beta = 0.11); participation was positively regressed on three exogenous determinants: age (beta = −0.12), years of education (beta = 0.21) and income (beta = 0.04). Additionally, the score of subjective characteristic of living place was positively regressed on the number of years of education (beta = 0.05) and on the number of household members (beta = 0.05), and a decrease in health status was positively regressed on age (beta = 0.29) and the number of household members (beta = 0.02) and negatively on years of education (beta = −0.07). Some links were created between social variables. Social networks were correlated with support (r = 0.50). Loneliness was regressed on social networks (beta = −0.26) and health status (beta = 0.17); both participation and trust were regressed on support (beta = 0.15 and beta = 0.34, respectively), and subjective assessment of home environment was regressed on health status (beta = −0.10) (Fig. 2). Finally, our exogenous variable HRQoL was regressed on four social variables: social networks (beta = 0.11), social support (beta = 0.180), social participation (beta = 0.14) and negatively on loneliness (beta = −0.18). HRQoL was observed as correlated with social trust and health status (r = 0.17 and r = −0.49, respectively). Additionally, we observed a regression pathway from the number of years of education to HRQoL (beta = 0.14).

The last part of our analyses focused on differences between males and females. At first, five separate OLS models were done (covariates: age, ratio of individual’s household income to median household gross adjusted disposable income (per person per year) in current (2011) USD PPP, total number of people living in the household and total number of years of education) and the gender differences were observed for Courage Social Networks Index (CSNI) (p = 0.013); Social Support (Oslo 3 Support Scale) (p = 0.001) and Loneliness (UCLA Loneliness Scale) (p = 0.046) and were not observed for Social participation (p = 0.145) and Trust (p = 0.789). Then the effect of social determinants on HRQoL by gender and age groups was verified on pathway model (Fig. 2) and the results are presented in Table 3. The significant interaction between the effects of age and social support, as well as age and social participation on HRQoL, was found. The role of social networks, social support, participation, trust and loneliness in HRQoL was significant and the only difference between pre-elderly (aged 50–64) men and women was in the effect of social support (Oslo 3 Support Scale). The same change in social support has led to a higher increase in quality of life among men than in women (assuming other social determinants constant). The same phenomenon was observed in older (65–79 years) group. Additionally, in this group we observed a different role of social participation in HRQoL, but here the effect was greater in females than in males. A slightly different effect was observed in the older-old group, as there were no differences in the roles of these social dimensions which were observed in younger groups, but differed in a role of social networks, which was positively linked with HRQoL, stronger in men than in women (Table 3).

**Table 3:** Results from the path model. Path and correlation (for trust) coefficients, standard errors and p values for quality of life (WHOQOL-AGE) regressed on social determinants across gender and age groups and p values for the difference in path coefficients between genders

	50–64					65–79					80+				
	Male		Female		p^2^	Male		Female		p^2^	Male		Female		p^2^
	B (SE)	p^1^	B (SE)	p^1^		B (SE)	p^1^	B (SE)	p^1^		B (SE)	p^1^	B (SE)	p^1^	
Courage Social Networks Index (CSNI)	0.140 (0.026)	<0.001	0.124 (0.021)	<0.001	0.6467	0.083 (0.028)	0.003	0.116 (0.024)	<0.001	0.3661	0.142 (0.060)	0.019	0.003 (0.040)	0.938	0.0469
Social Support (Oslo 3 Support Scale)	0.194 (0.027)	<0.001	0.117 (0.021)	<0.001	0.0145	0.281 (0.028)	<0.001	0.161 (0.025)	<0.001	0.0028	0.179 (0.056)	0.001	0.254 (0.040)	<0.001	0.3532
Social participation	0.160 (0.022)	<0.001	0.135 (0.018)	<0.001	0.6397	0.185 (0.036)	<0.001	0.256 (0.024)	<0.001	0.0163	0.378 (0.060)	<0.001	0.273 (0.043)	<0.001	0.3765
Trust	0.171 (0.024)	<0.001	0.185 (0.021)	<0.001	0.9158	0.082 (0.029)	0.004	0.136 (0.026)	<0.001	0.1093	0.100 (0.053)	0.059	0.099 (0.039)	0.011	0.9691
Loneliness (UCLA Loneliness Scale)	−0.203 (0.026)	<0.001	−0.178 (0.021)	<0.001	0.1619	−0.142 (0.030)	<0.001	−0.152 (0.025)	<0.001	0.098	−0.237 (0.068)	<0.001	−0.165 (0.043)	<0.001	0.521
Model fit indexes															
RMSEA (95% CI)		0.062 (0.057; 0.066)					0.054 (0.049; 0.060)					0.051 (0.041; 0.062)			
CFI		0.919					0.941					0.944			

B—standardized path coefficient, SE—standard error for standardized path coefficient, p^1^—p value for significance of the path coefficients, p^2^—p value for gender differences in path coefficients

## Discussion

The study confirmed an effect of social determinants on HRQoL across age and gender groups which has been observed across different studies so far. One of the added values of our research is that the analyses account for multiple relations between analysed social determinants in the path model of direct and indirect relations. We analysed a role of social networks, social support, social participation, trust and loneliness in HRQoL by one model, which may add to the understanding of the whole process, and may explain inconsistencies observed in those studies, which tried to analyse each of these constructs separately. Although conceptually different, some of psychosocial dimensions analysed have much in common, and the use of multipathway modelling enabled us to account for that.

In our study, social networks were found as a significant determinant of HRQoL in almost all gender–age subcategories except old–old women. Our study did not show gender-related differences in the role of social networks on HRQoL in pre-elderly and elderly groups; however, differences were observed in old–old group showing an interaction effect of gender and social networks on HRQoL in this group.

Other studies showed a greater effect of social networks on HRQoL among women than men, which was not typically observed in our study. The effect of size of social networks on life satisfaction was stronger for women [58]. Similarly, the results of meta-analysis showed a greater effect of social networks on subjective well-being measured as life satisfaction or happiness in women than men [59]. Low level of perceived social support was related with poor self-rated health among women but not in men [60].

Different effects of social networks on HRQoL among the old–old may be explained by socio-emotional selectivity theory or by the convoy model [61]. The differences may be caused by greater investment in the maintenance of social ties among women [59]; older women maintain also more extensive social network than older men [62]. Besides, women had more active relations with their kid and kin network, what was more important for their emotional functioning [47]. Women maintain more emotionally intimate relationships than men, mobilize more social support during the period of stress and provide more frequent and more effective social support to others than men [25, 46, 63]. Additionally, females who were socially isolated had worse mental health, and this association was stronger in women reporting high level of home and work stressors [25], social conditions and distress [64]. Greater role of social networks in HRQoL observed among males in our study may be the effect of the nature of the investigation. We performed a cross-sectional research which showed relations between differences in social network and differences in HRQoL but not the development of networks and its influence on HRQoL. Men having lower values and higher variability of social networks demonstrated higher correlation coefficients and possibly benefited more from the change of social networks than women.

Social networks in our study were assessed by the COURAGE-SNI, which evaluates individual’s social relationships characterized by closeness and frequency of contacts, whereas social support was assessed by the Oslo Social Support Scale which evaluates the number of close confidants, sense of concern and interest and relationship to neighbours. Correlation analysis showed that the two dimensions, although close (to each other), assessed slightly different constructs (rho = 0.51). Our study showed that social support was associated with HRQoL of old males and old–old females. Gender-related differences were found to be significant in old and old–old groups.

Research investigating gender-related differences in the role of social support in HRQoL is rather limited. The longitudinal study performed among people aged 75+ in Germany showed a strong positive impact of social support on HRQoL in men, not in women [65]. Research from culturally different region among Brazilian older population (60+) found that the relationship between social support and SRH differed between older men and women [60]. A moderating role of gender in the effect of positive social interaction and tangible support on life satisfaction was observed among older Malaysians [66].

Our study also showed that, beyond other social determinants considered, higher social participation is also a determinant of better quality of life across gender and age groups. However, gender-related differences were observed only in the age 65–79 category, showing that the same increase in social participation led to a higher increase in the HRQoL in women than in men. Comparatively, the results from the SHARE study revealed that social participation was positively associated with HRQoL and life satisfaction among participants aged 60–79 but not in the old–olds, what was explained by a fact that the old–olds were involved in lower number of activities and less frequently in pleasant activities [58, 61]. Brazilian studies showed a significant relation between participation in group activities and SRH in males, not in females [60]. This might be associated with engagement in various types of social participation: collective, productive and political by gender groups. The main aim of collective participation is spending time together, in case of productive participation it is rendering of goods, services and benefits for others, finally political participation mainly aims being involved in decision making acts about social groups. The studies showed more frequent engagement in political activities, clubs or paid work outside home in men, while women are more likely to be engaged in takeing care, doing more volunteer work and caregiving outside the home. Besides, higher level of social participation among older men may be explained by higher level of physical functioning, education or better occupational career than women [67].

Our study also found a correlation between trust and HRQoL in pre-elderly and elderly males and females and old females. Similarly, Tokuda observed that greater interpersonal trust was related with better HRQoL [68]. The ELSA study showed that HRQoL increases with trusting relationships with children, family and friends. Interpersonal trust has also been recognized as a stronger predictor of survival in women than in men [49].

Finally, in the presented study the effect of loneliness on HRQoL was observed in all groups; however, no gender-related differences in the effects were noticed. To our knowledge, there are no studies investigating gender-related differences in the role of loneliness in quality of life, although it seems to be natural that especially older individuals experiencing feelings of loneliness have worse their quality of life as it was observed in the cross-sectional Swedish study performed among people aged 75+ [69].

The current study has several strengths. The main benefit is the knowledge about gender-related roles and the magnitude of the effects of different social determinants on HRQoL of older adults (which was accounted for other social determinants, functional status and subjective assessment of -home build environment). Other strengths include the following: (1) relatively large sample size from the three countries from different European regions, which give the possibility to compare the results from three age groups, taking into account also the oldest (80+) group of people; (2) structural equation modelling used to verify relations between different determinants of HRQoL by multi-pathway modeling and enabling adjustment for several covariates; (3) HRQoL was measured by questions specific to the older age and in order to measure social networks we used a universal tool built for measuring social networks across different European countries and age groups: the COURAGE-SNI [52]. Our study has also some limitations; as the study design was cross-sectional, it was not possible to infer causality. Consequently, we may conclude that individuals with higher social dimensions demonstrated higher quality of life; however, the study does not provide evidence for the effectiveness of population interventions in the area of social determinants. The study was performed among noninstitutionalized individuals, thus those with the worst functional status or quality of life were rather unlikely to be included in the study; the purpose of our study, however, was to assess the role of social determinants which are present among older adults in general population. Another limitation is related to the fact that variables such as HRQoL and social determinants were both based on self-reported data as well as it is likely that factors such as personality, optimism or pessimism bias the findings, although existing data did not confirm gender-related differences in association between personality characteristic and health outcomes [70–72]. The response rate in the COURAGE project ranged from 53 to 70%, and therefore there was a possibility of sample selection bias; however, we believe that it is less likely as the relative differences between un-weighted and population-weighted variables represented general characteristics <5%. Finally, effects observed in European region may not match regions and countries with different cultural and social circumstances.

In summary, our research showed gender-related differences in the effects of social determinants on quality of life of older adults showing those differences in the effect of social networks in 80+, social support in 50–64 and 65–79, and social participation in 65–79 age category. New generations of older adults who achieved the Third or Four age presented different experiences coming from previous stages in relation to gender-related differences. Social characteristics of women and men belonging to the younger cohorts in comparison to older ones indicate wider social and cultural changes observed in societies in relation to social position, social expectations and aspirations of current women and men. The knowledge of the role of social determinants and various influences in these roles across gender groups may provide useful information on how to shape social interventions at the population level to improve quality of life of older adults, which would help achieve successful ageing. Our study showed some directions of possible social intervention, focusing on the improvement of social participation in different forms of social activity especially in older women. Further well-designed, follow-up studies are required to address a question about the type of the intervention and the expected effect.

## Electronic supplementary material

Below is the link to the electronic supplementary material.

Supplementary material 1 (DOCX 342 KB)
