# Supplementary material for: Gender-related differences in the multi-pathway effect of social determinants on quality of life in older age—the COURAGE in Europe project
Source: Qual Life Res. 2017 Mar 3;26(7):1865–78. doi: 10.1007/s11136-017-1530-8 (PMC5486906; doi:10.1007/s11136-017-1530-8)
Supplement: Supplementary file 1 — Supplementary material 1 (DOCX 342 KB) [file 11136_2017_1530_MOESM1_ESM.docx]

**Title:** Gender-related differences in the multi-pathway effect of social determinants on quality of life in older age - the COURAGE in Europe project

**Journal:** Quality of Life Research

**Author names:** Beata Tobiasz-Adamczyk, Aleksander Galas, Katarzyna Zawisza, Somnath Chatterji, Josep Maria Haro,

José Luis Ayuso-Mateos, Seppo Koskinen, Matilde Leonardi

**Corresponding author:**

Beata Tobiasz-Adamczyk, Department of Medical Sociology, Chair of Epidemiology and Preventive Medicine, Jagiellonian University Medical College, Kopernika 7a, 31-034 Krakow, Poland. e-mail address: mytobias@cyf-kr.edu.pl

Figure 1. Path model specifying association between social and demographic, living place, health related determinants and quality of life.

Social Networks Index (COURAGE-SNI)

Quality of life (WHOQOL-AGE)

Self-rated health

Total number of people living in the household

Social Support (Oslo 3 Support Scale)

Social participation

Trust

Loneliness (UCLA Loneliness Scale)

Level of education

Household income per person per year

Health and disease scale (WHODAS II)

Married or living with partner

Subjective characteristic of living place/home (CBE-SR-Home)

*

*

*

*

*

*

*

Note: Level of education, marital status, total number of people living in the household and health and disease scale were regressed on age. Bold pathways were tested for the gender differences across age groups. *Pathway created as a consequence of modification indices values. Model fit indexes: RMSEA=0.064; CFI=0.918.

Figure 2. The following changes were made: The path model specifying association between social and demographic, living place, health related determinants and quality of life. This model is built based on correlation analysis the paths were added if the value of the correlation coefficients was greater than 0.3 ; RMSEA=0.081; CFI=0.820

Note: yrs_edu - numbers of years of education; income - household gross adjusted disposable income per capita; home_ch - total number of people living in the household; health - Health and disease scale (WHODAS II); networks - Social Networks Index (COURAGE-SNI); support - Oslo 3 Support Scale; loneline - UCLA Loneliness Scale; particip – participation; qol - Quality of life (WHOQOL-AGE)

Figure 3. The path model specifying association between social and demographic, living place, health related determinants and quality of life. This model was built based on correlation analysis (as in Fig. 2) and based on the values of modification indices, provided theoretical justifications; RMSEA=0.049; CFI=0.953

Note: yrs_edu - numbers of years of education; income - household gross adjusted disposable income per capita; home_ch - total number of people living in the household; health - Health and disease scale (WHODAS II); networks - Social Networks Index (COURAGE-SNI); support - Oslo 3 Support Scale; loneline - UCLA Loneliness Scale; particip – participation; qol - Quality of life (WHOQOL-AGE)

Figure 4. Path model. Demographic and economic variables were considered as exogenous predictors, social determinants as mediators and quality of life as endogenous outcome, RMSEA=0.169; CFI=0.583;

Note: yrs_edu - numbers of years of education; income - household gross adjusted disposable income per capita; home_ch - total number of people living in the household; health - Health and disease scale (WHODAS II); networks - Social Networks Index (COURAGE-SNI); support - Oslo 3 Support Scale; loneline - UCLA Loneliness Scale; particip – participation; qol - Quality of life (WHOQOL-AGE)

Figure 5. Path model as in Fig. 4. but demographic variables were put into the model as exogenous predictors in case of the value of correlation between demographic and other variables higher or equal 0.1. RMSEA=0.137; CFI=0.569;

Note: yrs_edu - numbers of years of education; income - household gross adjusted disposable income per capita; home_ch - total number of people living in the household; health - Health and disease scale (WHODAS II); networks - Social Networks Index (COURAGE-SNI); support - Oslo 3 Support Scale; loneline - UCLA Loneliness Scale; particip – participation; qol - Quality of life (WHOQOL-AGE)

Figure 6. Path model as in Fig. 5. With added paths based on the values of modification indices, provided theoretical justifications. Model included in the main manuscript. RMSEA=0.058; CFI=0.939

Note: yrs_edu - numbers of years of education; income - household gross adjusted disposable income per capita; home_ch - total number of people living in the household; health - Health and disease scale (WHODAS II); networks - Social Networks Index (COURAGE-SNI); support - Oslo 3 Support Scale; loneline - UCLA Loneliness Scale; particip – participation; qol - Quality of life (WHOQOL-AGE)

Table 1. R square for model presented on Fig. 6. for endogenous variables.

|  | R^2^ for whole model |
| --- | --- |
| Quality of life (WHOQOL-AGE) | 0.310 |
| Courage Social Networks Index (CSNI) | 0.065 |
| Social Support (Oslo 3 Support Scale) | 0.009 |
| Social participation | 0.105 |
| Trust (correlation) | 0.129 |
| Loneliness (UCLA Loneliness Scale) | 0.153 |
| Subjective characteristic of living place/home (CBE-SR-Home) | 0.034 |
| Health and disease scale (WHODAS II) | 0.438 |
